# The incidence, clearance and persistence of non-cervical human papillomavirus infections: a systematic review of the literature

**DOI:** 10.1186/s12879-016-1633-9

**Published:** 2016-06-14

**Authors:** Sylvia Taylor, Eveline Bunge, Marina Bakker, Xavier Castellsagué

**Affiliations:** GSK Vaccines, 20, Avenue Fleming, Parc de la Noire Epine, B-1300 Wavre, Belgium; Pallas Health Research and Consultancy BV, Rotterdam, The Netherlands; Cancer Epidemiology Research Program, Catalan Institute of Oncology (ICO), IDIBELL, L’Hospitalet de Llobregat, Catalonia, Spain; CIBER Epidemiología y Salud Pública (CIBERESP), Barcelona, Spain

**Keywords:** Human papillomavirus, Cervical, Penile, Oral, Vaginal, Systematic review, Incidence, Persistence, Clearance

## Abstract

**Background:**

Human papillomavirus (HPV) vaccines were designed to prevent cervical cancer in women and their provision remains a major public health need. However, HPV is also a major cause of non-cervical anogenital and oropharyngeal cancers and the potential benefit of vaccination likely extends beyond cervical cancer.

**Methods:**

A systematic literature search of PubMed (1995–2014) identified publications assessing the incidence, persistence, and clearance of non-cervical anogenital/oral HPV infections. Comparability with cervical HPV was assessed by identifying articles assessing the same or similar populations.

**Results:**

Available data suggest high incidence rates of non-cervical HPV infection in men and women, with HPV-16 predominating in all sites. The incidence of high risk HPV per 100 person-years ranged from 11.4 to 72.9 for penile infections, 6.7–47.9 at other male genital sites, and 4.4–36.7 and 5.3–23.4 for anal infections in men and women, respectively. The incidence per 100 person-years of oral infection with any HPV type ranged from 5.7 to 6.7 in men and 6.8–39.6 in women. Within the limitations of the data, there was a general pattern of higher incidence and clearance of non-cervical genital HPV infections, compared to cervical infections. HIV status, circumcision, number of sex partners and partner HPV status significantly influenced high-risk HPV incidence/clearance at male anogenital sites. Few studies assessed risk factors for oral HPV.

**Conclusions:**

Parallels appear to exist between the epidemiology of cervical and non-cervical HPV infections in terms of incidence, HPV-type distribution, and risk factors for infection. Available data suggest that non-cervical genital HPV infections may occur more frequently, with higher clearance rates, than cervical infections. More extensive studies could provide useful information for estimating vaccine impact, the wider cost-benefit of HPV vaccination, and guiding vaccination policy.

**Trial registration:**

Not applicable, as systematic review of the literature.

**Electronic supplementary material:**

The online version of this article (doi:10.1186/s12879-016-1633-9) contains supplementary material, which is available to authorized users.

## Background

Cervical cancer is the fourth most common cancer affecting women worldwide, with an estimated 528,000 new cases and 266,000 related deaths in 2012 [1]. Cervical cancer develops following persistent infection with oncogenic (or high risk) types of the human papillomavirus (HPV), that includes types 16, 18, 31, 33, 35, 39, 45, 51, 52, 56, 58, 59, 66 [2]. Approximately 70 % of cervical cancers are caused by HPV types 16 and 18, which along with several other high risk HPV types (HR-HPV), can be prevented by vaccination. However, the cancer-causing effects of HPV are not limited to the cervix; an estimated 50 % of penile, 88 % of anal, 43 % of vulvar, 70 % of vaginal, and 13–56 % of oropharyngeal cancers are attributable to HPV, primarily HPV-16 typically followed by HPV-18 [3, 4]. Sharply increasing trends in HPV-related oropharyngeal cancers have been observed in some countries [5]. The incidence of cervical cancer is far higher than that of non-genital or oropharyngeal cancers, and the provision of HPV vaccines to prevent cervical cancer remains a public health priority. However, together non-cervical and oropharyngeal cancers represented approximately 80,000 new HPV-related cancer cases worldwide in 2008, also signifying an important public health burden [6].

Available evidence from clinical trials indicates that current HPV vaccines can prevent vulval and vaginal and anal HPV infections, anogenital pre-cancers, and oral HPV infections [7] in women, and oral and anogenital infections and pre-cancer in men [7–14]. However, compared to vaccine efficacy/effectiveness data for cervical HPV infections and high-grade lesions, similar data for non-cervical HPV infections and lesions are scant. Moreover, our ability to measure the population level impact of HPV vaccination on non-cervical cancers is severely limited by a lack of systematic screening for non-cervical infections and pre-cancers, the relative rarity of these cancers, and for some, the absence of precursor lesions amenable to screening. The need to build ways of collecting such data nonetheless remains important to understand the full value of HPV vaccination, including cost-benefit, so that proper guidance can be given to vaccination policy. To this end, we conducted a systematic literature review to investigate the natural history of non-cervical HPV infections, and to identify parallels between the epidemiology of these infections and that of cervical infection. These data can inform on the design and scope of future studies of HPV vaccine effectiveness, aid in the interpretation of surveillance data, and point to knowledge gaps where further investigations may be warranted.

## Methods

### Objectives

The study objectives were to conduct a systematic review of the literature to describe the incidence, clearance and persistence of non-cervical HPV infections, including comparisons to cervical infections for which data were available for the same/or similar study population. We also examined risk factors for incident and persistent non-cervical HPV infections.

### Search strategy and selection criteria

A systematic review of the literature in PubMed used three search strings (Supplementary material) to identify non-cervical anogenital and oral HPV infections and was limited to studies published in English between 01 January 1995 and 12 July 2014. We used specific searches to obtain articles on cervical HPV infections reporting comparable estimates of incidence, clearance, or persistence in the same or similar populations and using comparable HPV testing methods as in the articles on non-cervical HPV infections. Articles were included if they contained information on incidence, persistence, clearance or duration of non-cervical HPV infections, risk factors for non-cervical HPV infections or risk groups for incidence of non-cervical infections. Articles were excluded if they reported diagnostic test research, if they were case studies, letters to the editor, editorials or comments, literature reviews, or meta-analyses presenting no original data. Articles were also excluded if no information was listed in the inclusion criteria, or if they reported data based on testing of self-collected vaginal samples only. PRISMA guidelines were followed for the report. Independent review in duplicate was undertaken for 30 % of screened titles and abstracts and for 10 % of full text articles. Article selection, data extraction, assessment of risk factors and quality control procedures are provided in Additional file 1.

During the search for cervical cohorts, studies were found which used self-collected vaginal samples but in which the samples were referred to as ‘cervical’ swabs. These studies were not identified with the original non-cervical search string that did not include the term ‘cervical’. We did not include studies when vaginal samples had been self-collected, because the original search may have missed studies that assessed self-collected vaginal samples, and because we could not be certain that they represented vaginal-only infections. For similar reasons cervical samples collected using cervical vaginal lavage in D’Souza et al. [15] were also excluded because we could not be certain that they represented cervical-only infections.

For ease of comparability, incidence, clearance and persistence rates were recalculated, if not provided, per 100 person-years, and all estimates of median (or mean if median not reported) time-to-clearance (or duration of infection) were calculated in terms of months. For example, conversion from 1000 person-months to 100 person-years values was done by multiplying by 12 and dividing by 10. All estimates of median time-to-clearance were calculated in terms of months (dividing days by 30.5). When confidence intervals (CI) were not already provided and there was sufficient data, we used the exact method to calculate 95 % CI for proportions.

Ethics approval was not required for this study.

### Definitions

The definition of HR-HPV was study specific. The HR-HPV types applicable to individual studies are provided in Additional file 2: Table S1.

Clearance, incidence, persistence, and duration of infection were defined broadly to capture the most studies: For individual HPV types (e.g., HPV-16), incidence was defined as at least one positive test for that HPV type following at least one negative test for that type. Clearance of infection was defined as a negative test for the individual HPV type following a positive test for that type.

Type-specific incidence of any HPV or HR-HPV was defined as a positive test for at least one HPV type or HR-HPV type not detected at baseline or other previous visit. For non-type-specific incidence, subjects were required to have tested negative for any HPV DNA or any HR-HPV DNA at baseline. We considered this to be non-type-specific incidence even if the HPV type(s) associated with the incident infection was known. In addition, for studies conducting multiple follow-up visits, incidence rates could be calculated by: 1) censoring subjects at the time of the first positive test (first acquired incidence); or 2) considering subjects to be at risk throughout the follow-up period and counting each visit where at least one new type was detected as an event (total acquired incidence).

Type-specific clearance of any HPV or HR-HPV was defined as a negative test for all HPV types or HR-HPV types detected at baseline (type-specific clearance of prevalent infection) or for all newly detected during follow-up (type-specific clearance of incident infection). Non-type-specific clearance was defined as at least one negative test for any HPV DNA or any HR-HPV DNA following a positive test for any HPV DNA or HR-HPV DNA at baseline or during follow-up.

Persistence was defined as at least two sequentially positive tests for at least one specific HPV type or HR-HPV type at least three months apart. Definitions for type-specific and non-type specific duration of infection followed those for clearance of infection.

For estimates related to any HPV and HR-HPV, calculations were required to be based on the total number of subjects rather than total number of infections as the denominator.

## Results

### Search results

We identified 38 articles from 25 unique study cohorts with relevant data on non-cervical anogenital or oral HPV infections (Table 1). A further 6 articles were identified that assessed cervical HPV infection in the same/similar populations. HPV-testing methods are summarised in Additional file 2: Table S1. Three articles were excluded due to unclear methods. Articles excluded due to improper testing methods or sample type were included under ‘no relevant data’.

**Table 1:** Summary of 38 articles included in the review

Author, study location (ref)	Study year	Population	Age	Number	Follow-up period (timing of follow-up visits)	Mean or median follow-up	Anatomical site	Outcomes (definition used for any/HR-HPV)^c^
Tobian, Uganda [19]	2003–2007	HIV + ve/-ve men	15–49	999	2 years (6, 12, 24 m)	Mean 14.4 m	Penile	Inc (TS, total) Clr (TS)
Kjaer, Denmark [63]	2000–2003	Military servicemen	18–19	374	6 m (6 m)	Range 5.4–7.8 m	Penile	Inc (NTS)
Backes, Kenya [17]	2002–2005	Uncircumcised men	17–24	966	1 year (6, 12 m)	Median 12.1 m	Penile	Inc (NTS, first)
Mbulawa, South Africa [22]	Not reported	HIV + ve/-ve men	19–67	486	2 years (6, 12, 18, 24 m)	-	Penile	Inc (TS, first) Clr (TS)^a^
Videla, Spain [23]	2005–2009	HIV + ve men	20- > 69	733	5 years (annual)	Median 24 m	Penile, anal, oral	Inc (NTS, first) Clr (NTS)
Wikström A, Sweden [21]	Not reported	MSW, sexually active without clinical HPV	18–54	235	(1–5 visits)	Mean 3.5 m between visits	Penile	Inc (TS) Clr/Pers (TS)
Gray, Uganda [18]	2003–2006	HIV-ve married men	15–49	840	24 m (6, 12, 24 m)	-	Penile	Inc Clr
Serwadda, Uganda [16]	2003–2007	HIV + ve men	15–49	210	24 m (24 m)	-	Penile	Inc (TS) Clr
Grabowski, Uganda [24]	2003–2007	HIV + ve/-ve men	15–49	936	24 m (6, 12, 24 m)	-	Penile	Pers (TS)
Giuliano, Brazil, Mexico, USA [53]	2005–2009	HIV-ve men	18–70	1159	4 years (every 6 m)	Median 27.5 m	Penile	Inc (NTS, first) Clr (TS)
Darwich, Spain [20]	2005–2009	HIV + ve men	20- > 69	606	5 years (annual)	Median 24 m	Penile, anal	Inc^b^ Clr
de Pokomandy, Canada [38]	2002–2007	HIV + ve MSM	21–66	247	3 years (every 6 m)	Mean 30.8 m	Anal	Inc Clr
Nyitray, Brazil, Mexico, USA [35]	2005–2009	Men	18–70	MSM:156 MSW:954	6 m (6 m)	Median 6.7 m	Anal	Inc (TS) Clr (TS) Pers (TS)
Goodman, Hawaii, USA [36]	1998–2003	Sexually active women	18–85	431	5 years (every 4 m)	Mean 16 m	Anal	Inc (NTS, first) Clr (TS)
Shvetsov, Hawaii [39]	1998–2003	Sexually active women	18–85	431	5 years (every 4 m)	Mean 16 m	Anal	Clr (TS)
Moscicki, USA [40]	1990- > 2004	Sexually active women	13–21	75	3–9.5 years (every 4 m)	Mean 84.5 m	Anal	Clr (TS, 2+ neg)
Mullins, USA [37]	1996–2001	HIV + ve and HIV-ve at risk	12–18	496	6 years (annual)	Mean 22.4 m	Anal	Inc (NTS, first)
Glick, USA [33]	2009–2010	MSM	16–30	94	1 year (every 6 m)	-	Anal	Inc (NTS, unclear) Clr (TS)
Hernandez, USA [34]	1998–2000	HIV + ve MSM	Mean 45	369	2 years (every 6 m)	-	Anal	Inc (TS, total)
Lu, Arizona USA [31]	2003–2005	Men	18–44	285	2 years (every 6 m)	Median 15.5 m	Male genital (shaft, coronal sulcus, glans, scrotum)	Inc (TS, total) Clr (TS)
Hernandez, Hawaii [59]	2004–2006	Circ, UnC men	18–79	357	2.5 years (every 2 m)	Mean 14 m	Male genital (coronal sulcus, glans, shaft, scrotum)	Clr (TS, 2+ neg)
Giuliano, Arizona USA [25]	2003–2005	Men	18–44	290	2 years (every 6 m)	Mean 15.5 m	Male genital (coronal sulcus, glans, shaft, scrotum)	Inc (NTS, first & total) Pers (NTS) Clr (NTS)
Morales, Mexico [26]	2003–2004	MSW	Median 36	351	3 years (every 4 m)	Median 19.8 m	Male genital (scrotum, shaft, balano-preputial groove, urinary meatus)	Inc (NTS, unclear) Clr (TS)
Partridge, USA [29]	2003–2006	MSW university students	18–20	240	3 years (every 4 m)	Median 12.9 m	Male genital (glans &urethral meatus, shaft, scrotum)	Inc (NTS, first)
Lajous, Mexio [27]	2000–2003	Male soldiers	16–40	336	1 year (1 year)	-	Male genital (shaft, coronal sulcus, scrotum urethral meatus)	Inc (NTS) Clr (TS) Pers (TS)
Albero, Brazil, Mexico, USA [28]	2005–2009	HIV-ve men	18–70	4033	4 years (every 6 m)	Median 17.5 m	Male genital (shaft, coronal sulcus, glans, scrotum)	Inc (NTS, first) Clr (TS, 2+ neg)
Winer, USA [43]	1990–2000	Female university students	18–20	444	10 year (every 4 m)	Mean 41.2 m	Vulvovaginal	Inc (NTS, first)
Edelstein, USA [45]	2008–2010	Male university students	Median 20	212	1.5 years (every 4 m)	Median 10.7 m	Oral (rinse/OP swab)	Inc (NTS, first)
Kero, Finland [48]	Not reported	Male partners of pregnant women	19–46	131	7 years (2, 6, 12, 24, 36 m & 7 years)	Mean at visit 1: 1.8 m, visit 2: 5.9 m, visit 3: 12.1 m, visit 4: 24.7 m, visit 5: 36.8 m, visit 6: 77.0 m	Oral (scraping)	Inc
Kero, Finland [30]	2006–2008	Pregnant women and their male partners	Women: 19–46 Men: 20–52	46 women, 46 men	7 years (7 years)	-	Oral (scraping), male genital (penis/urethra)	Inc (NTS) Clr (TS)
Kero, Finland [46]	Not reported	Males partners of pregnant women	20–52	129 men	7 years (2, 6, 12, 24, 36, 7 years)	Mean in 74 HPV + ve 43.8 m	Oral (scraping)	Inc (NTS, first) Clr (TS)
Louvanto, Finland [51]	Not reported	Women with persistent cervical HPV (≥24 months) and HPV-ve controls	Mean 25.2–26.4	43 cases 52 controls	6 years (possibly annual)	Mean 65.2 m (cases) 38.4 m (controls)	Oral (scraping)	Clr(TS)
Rintala, Finland [50]	2006–2008	Pregnant women and their male partners	Women: 19–46 Men: 20–52	331 women, 131 men	2 years (2, 6, 12, 24 m)	Mean 26.9 m (women) 25.9 m (men)	Oral (scraping)	Inc (NTS, first) Clr (NTS)
Pickard, USA [49]	2009–2010	University students	18–30	1000	3 m (3 m)	-	Oral (rinse)	Inc (TS) Clr (TS) Pers (TS)
D’Souza, USA [15]	2004–2005	HIV + ve and high-risk HIV-ve adult women	Not reported	199	6 m (6 m)	-	Oral (rinse)	Inc (TS) Clr (NTS)
Kurose, Japan [52]	2000–2002	Healthy volunteers	3–85	662	2.5 years (2.5 years)	-	Oral (scraping)	Clr (TS) Pers (TS)
Darwich, Spain [47]	2005–2009	HIV + ve men	Median 41	733	5 years (annual)	Median 24 m	Oral (scraping/rinse)	Clr (TS)
Kreimer, Brazil, Mexico, USA [44]	2007–2009	HIV-ve men	18–70	1626	4 years (every 6 m)	Median 12.7 m	Oral (rinse)	Inc (NTS, first) Clr (TS)

*Abbreviations*: *IQR* interquartile range, *MSM* men who have sex with men, *MSW* men who have sex with women, *Inc* incidence, *Clr* clearance, *Pers* persistence, *Circ* circumcised, *UnC* uncircumcised, *OP* oropharyngeal

^a^If HPV types present-negative-present in sequential visits then negative result was considered false

^b^In calculation of incidence of individual HPV types, subjects were required to be negative for any HPV DNA at baseline at not just negative for that type

^c^Definitions for Any or Any HR-HPV infections: *TS* type specific. For incidence, defined as a positive test for at least one HPV type or HR type not detected at baseline (or other previous visit). For clearance, defined as a negative test for all HPV types or HR-HPV types detected at baseline or newly detected during follow-up. *NTS* non-type-specific. For incidence, defined as testing negative for any HPV DNA or any HR HPV DNA at baseline. For clearance, defined as at least one negative test for any HPV DNA or any HR-HPV DNA following a positive test at baseline or during follow-up. *2+ neg* 2+ negative test results required to define an infection as having cleared. *first* subjects censored at time of first positive test. *total* subjects not censored and each visit where new type(s) detected counted as event. *unclear* unclear if incidence based on firstacquired or total-acquired

Median follow-up times varied between 6.7 and 84.5 months, with all but 8 studies conducting at least two follow-up visits at intervals ranging between 2 and 12 months. Among the 31 studies reporting data on incidence of any HPV or any HR-HPV, 10 (32 %) used type-specific definitions which counted any newly detected type as an incident infection and 17 (55 %) used non-type-specific definitions that required subjects to be negative for any HPV or any HR-HPV at baseline; an additional 4 studies reported data on incidence of individual HPV types only. A total of 31 studies were known to have multiple follow-up visits. Among the 31, 20 assessed incidence of any HPV or HR-HPV: 13/20 (65 %) estimated incidence based on time to the first acquired infection; 3/20 (15 %) considered each new infection as an event; for 2/20 it was unclear (10 %); 1/20 (5 %) used both methods; and 1/20 (5 %) did not conduct any survival analysis. Among the 29 studies reporting data on HPV clearance, type-specific definitions were used more consistently, with only 5 (17 %) using non-type-specific definitions. Three studies (10 %) required at least two consecutive negative test results to define an infection as having cleared.

### Penile HPV infections

#### Incidence

In studies conducted in Africa, HR-HPV incidence was higher in human immunodeficiency virus positive (HIV + ve) men (42.0–72.9/100 person-years) than HIV negative (HIV-ve) men (19.7–32.9/100 person-years) (Table 2). Studies in Spain reported higher HPV incidence in HIV + ve men who have sex with men (MSM) than in men who have sex with women (MSW) (11.6 versus 5.1/100 person-years), but similar incidences of HPV-16 and HPV-18 infection. Genotype-specific incidence was highest for HPV-16 in at least one study subgroup in 7/9 studies (HIV + ve, HIV-ve, circumcised, uncircumcised and MSW) [8, 16–21].

**Table 2:** Incidence and clearance of penile and other male genital HPV infections

Author (ref)	Population, age (years)	Incidence					Clearance					
		Unit	Any HPV	HR	16	18	Type of Infection	Unit of clearance	Any HPV	HR	16	18
Penile												
Tobian [19]	HIV-ve, 15–49 HIV + ve,15–49	per 100py		32.9 (TS) (30.0–36.0) 66.5 (60.4–72.2)	4.2 (3.1–5.6) 10.4 (6.9–14.7)	3.7 (2.6–5.0) 8.0 (5.1–11.8)	Prevalent	per 100py		170.2 (TS) (156.7–184.4) 114.7 (103.3–129.1)	168.2 (130.0–213.9) 70.3 (47.1–101.0)	178.3 (125.6–245.8) 122.8 (83.4–174.3)
Gray [18]	HIV-ve I, 15–49 HIV-ve C, 15–49	per 100py	-	19.7 (15.3–24.9) 29.4 (24.6–34.9)	3.7 (1.0–6.3) 4.9 (3.1–7.6)	1.7 (0.6–3.7) 5.6 (3.6–8.4)	Prevalent	per 100py			190.9 (118.2–291.8) 167.6 (113.9–237.8)	285.7 (158.4–466.8) 157.9 (88.4–260.4)
Serwadda [16]	HIV + ve I, 15–49 HIV + ve C, 15–49	% at 24 m	-	42.0 (TS) (31.1–55.5) 57.0 (46.3–67.2)	5.8 (1.6–14.2) 14.9 (7.7–25.0)	4.3 (0.9–12.0) 11.1 (5.2–20.1)	Prevalent	% at 24 m			66.7 (34.9–90.1) 63.2 (38.4–83.7)	72.7 (39.0–94.0) 91.7 (61.5–99.8)
Kjaer [63]	Military, 18–29	% at 6 m	13.8 (NTS) (8.6–19.0)	-	-	-	-	-	-	-	-	-
Backes [17]	UnC, 17–24	per 100py	All: 59.2 (NTS, first) (51.6–67.6) Glans: 53.3 (46.1–60.5) Shaft: 25.9 (22.3–30.0)	All: 37.3 (32.0–43.2) Glans: 32.8 (28.1–38.0) Shaft: 15.7 (13.1–18.7)	All: 13.1 (10.7–15.6) Glans: 10.3 (8.3–12.7) Shaft: 5.3 (3.8–7.0)	All: 4.3 (3.1–5.9) Glans: 3.7 (2.6–5.3) Shaft: 1.8 (1.1–2.9)	-	-	-	-	-	-
Mbulawa [22]	All, 18–66 HIV-ve, 18–66 HIV + ve 18–66	per 100py	66.8 (TS, first) (52.3–83.0) 62.3 (47.2–79.5) 96.5 (57.1–163.2)	35.7 (28.1–45.4) 29.2 (21.9–39.1) 72.9 (47.2–112.8)	3.8 (2.2–6.5)	4.0 (2.4–6.8)	Incident	per 100py	114.1 (TS) (100.0–129.7) 128.2 (115.4–141.9) 96.4 (83.8–110.1)	123.9 (106.2–144.7)	121.5 (74.8–197.4)	160.0 (109.4–234.0)
Darwich [20], Videla [23]	HIV + ve MSM, 20- > 69 HIV + ve MSW, 20- > 69	per 100py	11.6 (NTS, first) (8.8–14.9) 5.1 (2.4–9.4)	-	1.7 (0.8–2.9) 1.4 (0.4–3.7)	0.4 (0.1–1.1) 0.4 (−)	Prevalent	per 100py	27.1 (NTS) (20.0–35.6) 23.6 (14.8–35.6)	-	29.4 (15.1–51.4) 29.5 (10.8–64.2)	40.0 (8.2–116.9) 14.6 (−)
Wikström [21]	MSW, 18–54	% at 3.5m^a^	17.1 (TS) (9.4–27.5)	11.4 (5.6–19.9)	4.5 (1.3–11.2)	0 (0–4.1)	Prevalent	% at 3.5m^a^-	41.7 (TS) (15.2–72.3)	42.9 (9.9–81.6	20 (0.5–71.6)	100 (1 case only)
Giuliano [53]	HIV-ve, 18–70	per 100py	46.1 (NTS, first) (41.2–51.6)	26.7 (23.8–29.9)	5.3 (4.3–6.4)	2.3 (1.7–3.0)	-	-	-	-	-	-
Genital												
Giuliano [25] (coronal sulcus, glans, shaft, scrotum)	Men, 18–44	per 100py	46.1 (NTS, Total) (41.8–61.2) 35.3 (NTS, first) (27.4–44.9)	25.0 (19.2–31.9) 18.6 (13.4–25.0)	5.8 (3.4–9.2)	1.0 (0.2–2.9)	Prevalent	% at 6 m % at 12 m % at 18 m	55.5 (NTS) 74.8 89.3	58.3 81.0 100	-	-
Morales [26] (scrotum, shaft, balano-preputial groove, urinary meatus)	MSW, median 36	per 100py	14.8 (NTS, unclear) (11.8–18.2)	6.7 (4.9–8.9)	1.7 (0.8–2.8)	0.4 (0.1–1.1)	-	-	-	-	-	-
Partridge [29] (glans &urethral meatus, shaft, scrotum)	MSW, 18–20	% at 24 m	62.4 (NTS, first) (52.6–72.2)	47.9 (38.6–58.0)	19.5 (14.0–27.2)	7.5 (4.1–13.6)	-	-	-	-	-	-
Lajous [27] (shaft, coronal sulcus, scrotum urethral meatus)	Male soldiers, 16–40	per 100py	21.5 (NTS) (15.6–28.7)	14.3 (9.8–19.9)	2.8 (1.3–5.3)	1.6 (0.5–3.6)	Prevalent	% at 12 m	70.6 (TS) (61.9–78.4)	69.0 (59.0–77.9)	68.8 (41.3–89.0)	100 (66.4–100)
Albero [28] (shaft, coronal sulcus, glans, scrotum)	HIV-ve men UnC, 18–70 HIV-ve men Circ, 18–70	per 100py	50.5 (NTS, first) (46.4–54.9) 45.6 (41–50.5)	28.4 (26.2–30.8) 28.7 (25.9–31.8)	5.8 (5–6.6) 6.6 (5.6–7.8)	2.5 (2–3) 2.9 (2.3–3.7)	-	-	-	-	-	-
Kero [30] (penis/urethra)	Male partners of pregnant women, 20–52	% at 7 years	32.3 (NTS) (16.7–51.4)	-	-	-	Prevalent	% at 7 years	88.9 % (TS) (51.8–99.7)	-	-	-

*Abbreviations*: *MSM* men who have sex with men, *MSW* men who have sex with women, *py* person-years, *UnC* Uncircumcised, *Circ* Circumcised, *m* months, *I* Intervention (immediate circumcision), *C* Control (circumcision delayed for 24 months), *TS* type-specific incidence defined as at least one positive test for any HPV type or HR-HPV type not detected at baseline, *NTS* non-type-specific incidence defined as at least one positive test for any HPV type or HR-HPV type among those negative for any HPV DNA or any HR-HPV DNA at baseline

^a^Mean interval between visits 3.5 m: Results at visit 1 and 2 considered here

In three articles that reported age-specific incidence of HPV infection the highest incidence was reported in the youngest age group studied (<30 or 15–24 year olds) [18, 19, 22].

#### Clearance and persistence

The rate of clearance of incident HR-HPV infection was 123.9/100 person-years (Table 2), with HPV-33 clearing most rapidly and HPV-58 least rapidly [22].

The clearance rate of prevalent penile infections ranged between 23.6 and 114.1/100 person-years for any HPV [19, 20, 23]. Clearance of HR-HPV was 114.7/100 person-years in HIV + ve men and 170.2/100 person-years in HIV-ve men [19]. HPV types tending to clear the least rapidly were HPV-52 and 16 in circumcised men, 52 and 58 in uncircumcised men over a 2-year period [18], 52 and 16 in HIV + ve MSM and 18 and 51 in HIV + ve MSW up to 5 years of follow-up [20], depending on study and population. Age-specific clearance rates were highest in men aged >30 years in two studies [19, 22].

**Fig. 1:**
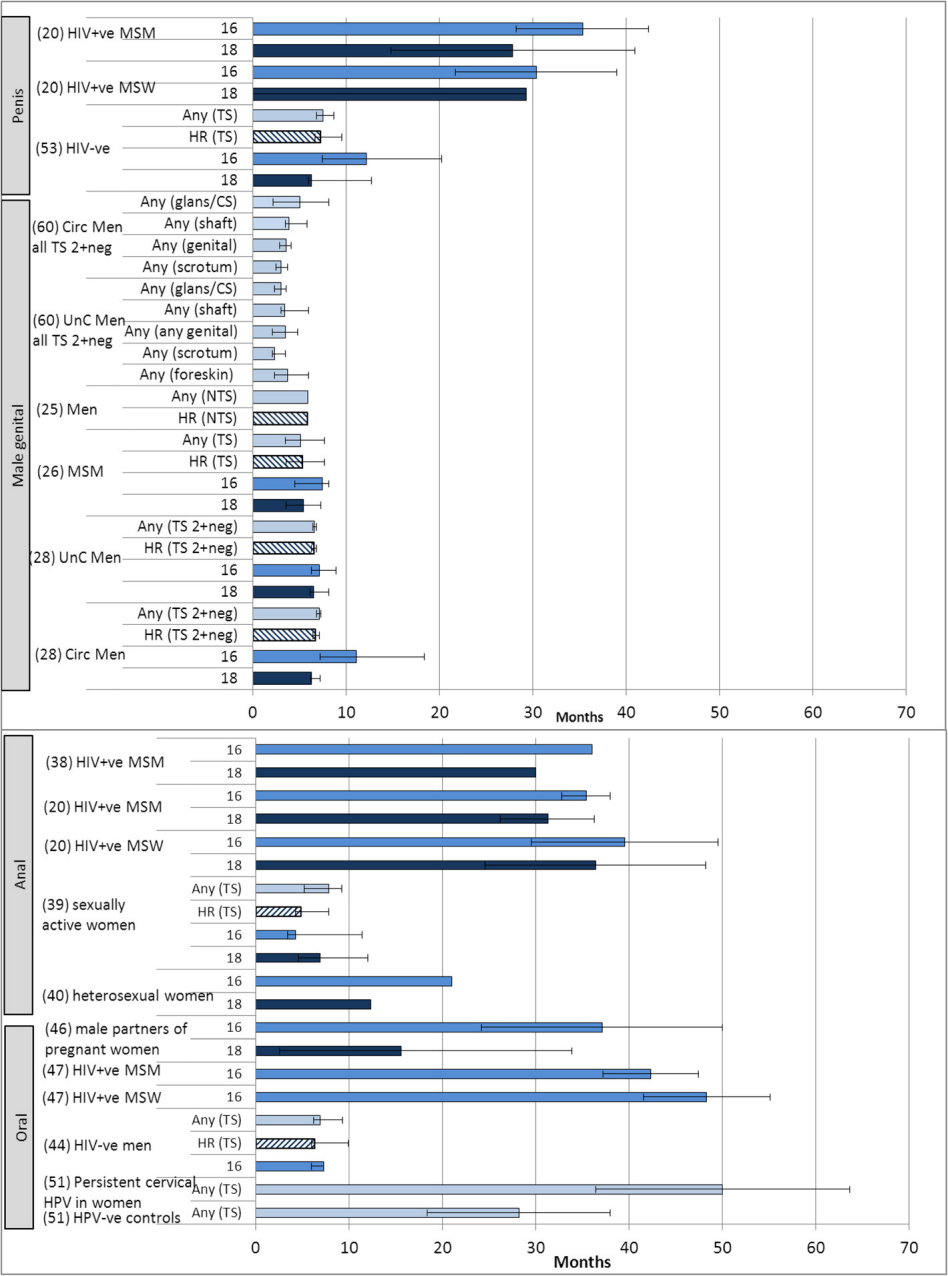
Median* Time to clearance (months, 95 % CIs) of HPV from non-cervical sites in men and women. Footnote: *mean reported for [20, 46, 47, 51]. *TS* type-specific incidence defined as at least one positive test for any HPV type or HR-HPV type not detected at baseline; *NTS* non-type-specific incidence defined as at least one positive test for any HPV type or HR-HPV type among those negative for any HPV DNA or any HR-HPV DNA at baseline

Time-to-clearance was 12.2 months for HPV-16 and 6.3 months for HPV-18 in HIV-ve men, versus 27.8–35.3 months for HPV-16 and 18 in HIV + ve men (Fig. 1).

HR-HPV persisted in 31 % of HIV-ve men and 43 % of HIV + ve men at 6 months, and in 25 %/28 %, respectively, at 12 months [24] (Table 3). Persistence of HR-HPV at 12 months was associated with higher HPV viral load at baseline in HIV + ve men [24].

**Table 3:** Type-specific persistence of HPV infections

					% (95 % CI) persistent at end of time frame			
Site	Reference	Population, age (years)	Infection type	Time-frame (months)	Any HPV	HR	16	18
Penile	Wikström; Sweden [21]	MSW, 18–54	Prevalent/ Incident	3.5	50 (23–77)	57 (18–90)	75 (19–99)	0 (0–98)
	Grabowski; Uganda [24]	HIV- men, 15–49 HIV+ men, 15–49	Prevalent Prevalent	6 12 24 6 12 24		31 (27–37) 25 (20–30) 12 (9–16) 43 (37–48) 28 (23–34) 29 (25–34)		
Anal	Nyitray; Brazil, Mexico, USA [35]	MSM, 18–70 MSW, 18–70	Prevalent Prevalent	6 6	59 (48–70) 32 (24–41)	51 (36–66) 24 (14–37)	73 (39–94) 0 (0–16)	38 (9–76) 0 (0–84)
Male Genital	Lajous; Mexico [27]	Male soldiers, 16–40	Prevalent	12	29.4 (21.6–38.2)	31.0 (22.1–41.0)	31.3 (11.0–58.7)	0 (0–33.6)
Oral	Pickard; USA [49]	Male/female students, 18–30	Prevalent	3	38 (19–59)	42 (15–72)	0 (0–98)	
	Kurose; Japan [52]	Male/female volunteers, 3–85	Prevalent	30	50 (9–93)	0 (0–98)	0 (0–98)	-

*Abbreviations*: *MSM* men who have sex with men, *MSW* men who have sex with women

#### Cervical infections in comparable populations

In South Africa, incidence and clearance of penile and cervical infections was assessed among 468 male–female couples aged 18–67 years (Table 4) [22]. Incidence and clearance rates were significantly higher among men compared with women (incidence of any HPV 66.8 versus 40.7/100 person-years; clearance of any HPV 114.1 versus 80.3/100 person-years). In regression analyses adjusting for HPV type, incidence rates remained higher in men when stratified by age-group and HIV status; clearance rates remained higher in men when stratified by age-group and among HIV + ve individuals (Table 4). HIV status was the only significant predictor of clearance in men and women, whereas in multivariate analyses, incidence was significantly predicted by HIV status and partner infection with same HPV type in men and women, and age at first sex and hormonal contraceptive use in women only.

**Table 4:** Comparison of non-cervical HPV infections and cervical HPV infections in same or similar study populations

Ref, site	Population, age (years)	Incidence (95 % CI)					Clearance (95 % CI)					
		Unit	Any	HR	16	18	Inf. type	Unit	Any	HR	16	18
Darwich; Spain; Penile [20]	HIV + ve MSM, 20- > 69	Per 100py	-	-	1.7 (0.8–2.9)	0.4 (0.1–1.1)	Prev	Per 100py	-	-	29.4 (15.1–51.4)	40.0 (8.2–116.9)
	HIV + ve MSW, 20- > 69	Per 100py	-	-	1.4 (0.4–3.7)	0.3 (ND)	Prev	Per 100py	-	-	29.5 (10.8–64.2)	14.6 (−)
Videla; Spain; Cervical [64]	HIV + ve women, 20–64	% at 36 m	-	-	9 (3.4–18.5)	7 (2.6–14.6)	Prev	% at 36 m	-	-	46 (26.6–66.6)	29 (3.7–71.0)
Comment: Study period 1999–2003 for women v. 2005–2009 for men												
Mbulawa; South Africa; Penile [22]	HIV + ve/-ve Men in a relationship, 19–67	Per 100py	66.8 (TS, first) (52.3–83.0)	35.7 (28.1–45.4)	3.8 (2.2–6.5)	4.0 (2.4–6.8)	Prev/Inc	Per 100py	114.1 (TS) (100–130.7)	124.9 (106.2–144.7)	121.5 (74.8–197.4)	160.0 (109.4–234.0)
Mbulawa; South Africa; Cervical [22]	HIV + ve/-ve Women in a relationship, 18–66	Per 100py	40.7 (TS) (31.7–52.2)	18.7 (13.8–25.7)	2.4 (1.3–4.7)	1.4 (0.6–3.3)	Prev/Inc	Per 100py	80.3 (TS) (68.4–94.2)	74.7 (61.3–91.4)	38.9 (16.7–90.5)	127.9 (85.2–94.2)
Comment: Incidence and clearance significantly higher in men v. women. In type- adjusted analyses, incidence remained higher in men by age group and HIV status; clearance remained higher in men by age group and HIV + ve (96.4 versus 66.0/100 person-years), but not HIV-ve (128.1 versus 132.1/100 person-years)												
Giuliano; USA (Arizona); Male genital [25]	Adult men, 18–44	Per 100py	46.1 (NTS, total) (41.8–61.2) 35.3 (NTS, first) (27.4–44.9)	25.0) (19.2–31.9) 18.6) (13.4–25.0)	5.3 (3.4–9.2)	1.0 (0.2–2.9)	-	-	-	-	-	-
Giuliano; USA (Arizona); Cervical [32]	Women attending gynecology clinic,18–35	Per 100py	35.3 (NTS, first) (24.7–48.8)	-	7.1 (3.7–12.1)	1.0 (0.1–3.5)	-	-	-	-	-	-
Comment: Study period 2003–2005 for men v. 1996–1999 for women; follow-up at 6, 12, and 24 months for men v. 4 and 10 months for women. Med time to clearance anal v. cervical: 5.9 m (5.7–6.1) v. ~9 m for any HPV; 5.8 m (5.5–6.1) v. 8.5 m for HR-HPV. PGMY09/11 assay used to detect types 61, 62, 64, 67, 69, 72, 81, IS39, and CP6108 in anal but not cervical samples												
Goodman; USA (Hawaii); Anal [36, 39]	Sexually active women, 18–85	Per 100py	56.3 (NTS, first) (48.5–65.0)	23.4 (19.2–28.3)	3.0 (1.8–4.8)	2.3 (1.2–3.9)	Inc	Per 100py	89.3 (TS) (67.8–115.4)	110.0 (83.3–142.4)	141.2 (73.0–246.7)	130.0 (47.6–282.8)
Goodman; USA (Hawaii); Cervical [41]	Sexually active women, 18–85	Per 100py	15.6 (NTS, first) (12.9–18.7)	11.1 (9.0–13.6)	2.1 (1.4–3.2)	0.6 (0.2–1.2)	Inc	Per 100py	85.1 (TS) (64.2–110.4)	92.0 (67.8–122.0)	86.4 (41.4–158.9)	69.7 (14.4–203.6)
Comment: 431 of 972 women in cervical analysis as per availability of anal sample, complete questionnaire data, and valid HPV testing results. Med time to clearance anal v. cervical: 7.8 m (5.2–9.2) v. 8.5 m (5.8–12.6) for any HPV; 4.9 m (4.3–8.0) v. 8.0 m (5.5–11.8) for HR HPV; 4.3 m (3.4–11.4) v. 9.5 m (4.4–23.8) for HPV16; 6.9 (4.6–12.0) v. 14.0 (4.8–ND) for HPV18												
Moscicki;USA; (California); Anal [40]	Sexually active women, 13–21	-	-	-	-	-	Prev	% at 12 m	-	29.7 (TS 2 + neg) (19.0–41.7)	-	-
Moscicki;USA (California); Cervical [42]	Sexually active women, 13–22	-	-	-	-	-	Prev	% at 12 m	-	~35 % (NTS)	-	-
Comment: Anal and cervical analyses conducted on two partially overlapping cohorts of 75 and 531 women, respectively. Clearance defined as time to first of 2 consecutive negative results; data for any HPV not presented as cervical assay detected only 5 of 19 low-risk types detected by anal infection assay. HPV types 68, 69, 73, and 82 defined as HR types in analysis of anal but not cervical infection; ~35 % clearance at 12 m for cervical infections estimated from provided Kaplan-Meier curves												
Winer; USA; Vulvovaginal [43]	Female university students, 18–20	100py	16.0 (NTS, first) (13.7–18.6)	-	-	-	-	-	-	-	-	-
Winer; USA; Cervical [43]	Female university students, 18–20	100py	12.7 (NTS, first) (10.8–14.9)	-	-	-	-	-	-	-	-	-
Comment: Separate vulvovaginal and cervical swabs collected from 444 initially HPV-DNA-negative women. 35.4 % of infections were present at both sites												
Kero, Finland, Male Genital [30]	Partners of pregnant women, 20–52	% at 7 years	32.3 (NTS) (16.7–51.4)	-	-	-	Prev	% at 7 years	90 (TS) (55.5–99.7)	-	-	-
Kero; Finland; Oral [30]	Pregnant women, 19–46	% at 7 years	14.0 (NTS) (5.3–27.9)	-	-	-	Prev	% at 7 years	100 (TS) (29.2–100)	-	-	-
Kero; Finland; Cervical [30]	Pregnant women, 19–46	% at 7 years	16.7 (NTS) (6.4–32.8)	-	-	-	Prev	% at 7 years	70 (TS) (34.8–93.3)	-	-	-
Comment: Analysis based on only 46 men and 46 women returning for follow-up at 84 months. At year 7 the same infecting HPV genotype was detected in 11 % (95 % CI 0.3–48.2) and 30 % (6.7–65.2) of mean and women, respectively. HPV status during interval visits was not taken into account.												

Note: For male versus female comparison only date for penile/male genital infections versus cervical infections are presented

*Abbreviations*: *m* months, − data not provided, *TS* type-specific incidence defined as at least one positive test for any HPV type or HR-HPV type not detected at baseline, *NTS* non-type-specific incidence defined as at least one positive test for any HPV type or HR-HPV type among those negative for any HPV DNA or any HR-HPV DNA at baseline

### Male genital HPV infections

Male genital sites included samples from the scrotum, coronal sulcus or combined samples of both penile and non-penile sites (Table 2).

#### Incidence

Incidence of any HPV ranged from 14.8 to 50.51/100 person-years in adult men and military men [25–28]. Incidence in university students (median follow-up of 12.9 months) was 62.4 % [29]. Incidence in male partners of pregnant women was 32.3 % over 7 years after the initial assessment [30]. HPV-16 was the most frequently identified type in all studies, followed by 52 and 58 [25–27, 29], or 51 and 59 [28].

Two studies reported on age-specific incidences of male genital infections, of which one was restricted to 18 or 19 year olds [29]. In an adult population the highest incidence was observed in the oldest age group studied (41–44 years) for any HPV infection (55.6/100 person-years), but the highest incidence of HR-HPV was in 26–30 year-olds (33.1/100 person-years) [31].

#### Clearance and persistence

Median time-to-clearance of any HPV male genital infection was 3.5–7.1 months (Fig. 1). Time-to-clearance of HPV-16 was 7.1–11.1 months, and 5.4–6.5 months for HPV-18.

The median duration of incident male genital HPV infections ranged between 5.1 and 7.1 months [25, 26, 28]. The duration of HPV-16 and/or 18 infection ranged between 5.4 and 11.1 months [25, 26, 28]. In the United States (US), HR-HPV genital infections persisted in 42 % of men at 6 months, 19 % at 12 months and had cleared in all men at 18 months [25] (Table 2).

#### Cervical infections in comparable populations

Incidence of male genital and cervical infections was assessed in two similar but separate cohorts of men and women from Arizona (Table 4) [25, 32]. Incidence calculated based on the first acquired infection with any HPV was similar in men and women (35.3/100 person-years [95 %CI 27.4–44.9] versus 35.3/100 person-years [95 % CI 24.7–48.8]), but the cumulative incidence of infection by 12 months was substantially higher in men than in women (41 % versus 29 % [95 % CI 22–36] for any HPV; 32 % versus 19 % [95 % CI 13–25) for HR-HPV). Median time-to-clearance for penile compared with cervical infections was 5.9 months (95 % CI 5.7–6.1) versus approximately 9 months for any HPV, and 5.8 months (95 % CI 5.5–6.1) versus 8.5 months for HR-HPV. In Finland, where male genital and cervical infection data were collected at baseline and 7 years later among 46 pregnant women and their male partners, incidence and clearance of any HPV was also higher among men versus women: 32.3 % (95 % CI 16.7–51.4) versus 16.7 % (95 % CI 6.4–32.8) and 90 % (95 % CI 55.5–99.7) versus 70.0 % (95 % CI 34.8–93.3) (Table 4). Among men and women with genital HPV infections at baseline, the same infecting HPV genotype was present at the 7-year follow-up in 11 % (95 % CI 0.3–48.2) and 30 % (6.7–65.2), respectively [30].

### Anal HPV infections

Ten articles reported on anal HPV infections: seven reported infections in men and four in women (Table 5).

**Table 5:** Incidence and clearance of anal HPV infections

Author (ref)	Population, age (yrs)	Incidence (per 100py)				Clearance					
		Any HPV	HR	16	18	Type of Infection	Unit of clearance	Any HPV	HR	16	18
Males											
de Pokomandy [38]	HIV + ve MSM, 21–66	-	-	13.0 (9.6–17.6)	5.3 (3.5–8.0)	Prevalent	Per 100py			14.6 (10.2–21.2)	24.5 (16.9–35.4)
Nyitray [35]	MSM, 18–70 MSW, 18–70	31.1 (TS) (16.6–53.2) 9.7 (7.2–12.8)	25.4 (14.5–41.2) 4.4 (2.9–6.6)	5.8 (1.9–13.4) 0.8 (0.2–2.0)	4.6 (1.2–11.6) 1.1 (0.4–2.3)	Prevalent	% at 6 m-	57.1 (TS) (50.1–63.8) 70.9 (63.5–77.5)	55.8 (44.1–67.2) 77.5 (66.0–86.5)	27.2 (6.0–61.0) 100 (83.9–100)	62.5 (24.5–91.5) 100 (15.8–100)
Videla [23]	HIV + ve MSM, 20- > 69 HIV + ve MSW, 20- > 69	32.4 (NTS) (23.3–43.7) 7.9 (4.9–15.7)	-	-	-	Prevalent	Per 100py	12.6 (NTS) (10.2–15.2) 18.4 (12.2–26.5)	-	-	-
Darwich [20]	HIV + ve MSM, 20- > 69 HIV + ve MSW, 20- > 69	-	-	7.1 (5.5–9.5) 5.3 (3.0–8.6)	3.6 (3.5–5.2) 0.4 (0.1–1.8)	Prevalent	Per 100py	-		22.4 (17.6–28.1) 18.6 (7.4–38.2)	31.0 (20.3–45.4) 30.0 (7.8–76.8)
Mullins [37]	HIV-ve, 12–18 HIV + ve, 12–18	24 (NTS, first) (11–52) 40 (27–61)	11 (3.4–33) 27 (17–44)	-	-	-	-	-	-	-	-
Glick [33]	MSM, 16–30	46.2 (NTS, unclear) (45.6–46.9)	36.7 (36.2–37.3)	12.4 (12.1–12.6)	5.5 (5.3–5.6)	Prevalent	% at 1 year	81.3 (TS) (67.4–91.1)	66.7 (49.0–81.4)	50.0 (26.0–74.0)	66.7 (22.3–95.7)
Hernandez [34]	HIV + ve MSM, mean 45	21.3 (TS, total) (17.7–25.4)	13.3 (10.5–16.6)	3.5 (1.8–6.1)	3.7 (2.1–6.1)	-	-	-	-	-	-
Females											
Goodman [36], Shvetsov [39]	Sexually active women, 18–44	56.3 (NTS, first) (48.5–65.0)	23.4 (19.2–28.3)	3.0 (1.8–4.8)	2.3 (1.2–3.9)	Incident	Per 100py	89.3 (TS) (67.8–115.4)	109.9 (83.3–142.4)	141.2 (73.0–246.7)	130.0 (47.6–282.8)
Moscicki [40]	Sexually active women, 13–21	-	-	-	-	Prevalent	% at 12 m	-	29.7 (TS 2 + neg) (19.0–41.7)	45.0 (27.2–62.1)	55.6 (21.2–86.3)
Mullins [37]	HIV-ve women, 12–18 HIV + ve women, 12–18	14 (NTS,first) (9.2–22) 30 (24–38)	5.3 (2.6–11) 12 (8.4–16)	-	-	-	-	-	-	-	-

*Abbreviations*: *MSM* men who have sex with men, *MSW* men who have sex with women, *py* person-years, *TS* type-specific incidence defined as at least one positive test for any HPV type or HR-HPV type not detected at baseline, *NTS* non-type-specific incidence defined as at least one positive test for any HPV type or HR-HPV type among those negative for any HPV DNA or any HR-HPV DNA at baseline

#### Incidence in men

The incidence of anal HPV infection was higher in HIV + ve men (gender of partner not specified) and HIV-ve MSM (range 21.3–46.2/100 person-years), than in HIV + ve MSW (7.9/100 person-years) and MSW (HIV status not specified, 9.7/100 person-years). Among MSM, HPV-16 and 18 were among the HPV types with the highest incidence, although depending on the study, the incidences of HPV-51, 52 or 59 were similar or higher [33–35].

#### Incidence in women

In women, anal HPV incidence ranged from 14–56.3/100 person-years. HPV-16 and 52 were the most frequently infecting types [36]. The incidence of any and HR anal HPV infection in 12–18 year olds was higher in men than women (HIV-ve and HIV + ve), although the 95%CIs overlapped (Table 5) [37]. By contrast, in another study in women [36] the incidence (per 100 person-years) of any anal HPV infection was higher (56.3) than in all studies reported in male populations (range 7.9–46.2) (Table 5). In this study the incidence (per 100 person-years) of HR-HPV infection in women (23.4) was higher than that reported in MSW (4.4) and within the range observed in HIV + ve men or MSM (13.3–36.7).

A regression analysis conducted in a US study of sexually active women noted a statistically significant inverse relationship between acquisition of a new HR-HPV anal infection and age; with a 57 % (95 % CI 19–77) lower risk of acquisition among older women (≥45 years of age at baseline) than among younger women (<25 years at baseline) [36].

#### Clearance and persistence in men and women

Seven articles reported on the clearance rate of anal infections, of which five were in men who were HIV + ve and/or MSM, and two were in women (Table 5). The clearance rate of prevalent anal HPV infections in men varied between 14.6 and 66.7/100 person-years for specific HPV types. Amongst HR-HPV, the clearance rate was lowest for HPV-16 in 5/6 populations [20, 33, 35, 38]. For HPV-16 and 18 the time-to-clearance ranged between 30 and 39.5 months (Fig. 1).

In women, the clearance rate of incident anal HPV infections was 89.3/100 person-years, with a median time-to-clearance of 7.8 months [39] (Fig. 1). In another study, 56.5 % of women cleared any HR-HPV infection by year 3 [40]. In the single study that reported clearance of prevalent infections, HPV clearance in women appeared to be within the range reported in studies in men (Table 5).

HR-HPV anal infections persisted at 6 months in 51.0 % of MSW and in 24.2 % of MSM who were HR-HPV positive at baseline [35] (Table 3).

#### Cervical infections in comparable populations

In Hawaii, anal and cervical samples were collected in sexually active women aged 18–85 years every 4 months for an average of approximately 1.3 years [36, 39, 41]. Incidence (per 100 person-years) of anal versus cervical infections was more than three-fold higher for any HPV (56.3 [95 % CI 48.5–65.0] versus 15.6 [95 % CI 12.9–18.7]) and more than two-fold higher for HR-HPV (23.4 [95 % CI 19.2–28.3] versus 11.1 [95 % CI 9.0–13.6]); differences were similar for HPV-16 and 18 infections but not statistically significant (Table 4). Baseline cervical HPV status was not a significant risk factor for incidence of anal HPV infection. In contrast, clearance (per 100 person-years) did not significantly differ between anal versus cervical infections with either any HPV (89.3 [95 % CI 67.8–115.4] versus 85.1 [95 % CI 64.2–110.4], respectively) or with HR-HPV (109.9 [95 % CI 83.3–142.4] versus 92.0 [95 % CI 67.8–122.0], respectively).

In two partially overlapping cohorts of sexually active teenager and young adults in the US, clearance by 12 months was also similar for anal versus cervical infections (29.7 % [95 % CI 19.0–41.7] and approximately 35 %, respectively) [40, 42]. In the Hawaii cohort, there was some indication of higher clearance of anal than cervical HPV-16 and 18 infections, with more than two-fold differences in the median duration of infection (4.3 versus 9.8 months, and 6.9 versus 14 months, respectively), but CIs overlapped substantially (data not shown) [36, 39, 41].

### Vaginal HPV infections

We identified one study of vulvovaginal HPV infections that employed physician-collected swabs [43]. The incidence rate for vulvovaginal HPV infections among university students was 16.0 per 100 person-years (95 % CI 13.7–18.6) compared with 12.7 per 100 person-years (95 % CI 10.8–14.9) for cervical infections (Table 4). No information on clearance or persistence was available.

### Oral HPV infections

Eleven articles (considering six cohorts) reported on oral HPV infections: eight in men and five in women (Table 6).

**Table 6:** Incidence and clearance of oral HPV infections

Author (ref)	Population, age (years)	Incidence (95 % CI)					Clearance					
		Unit	Any HPV	HR	16	18	Infection type	Unit of clearance	Any HPV	HR	16	18
Males												
Edelstein [45]	Male students, median 20	% at 1y	12.3 % (NTS, first) (7.0–21.3)	-	0.8 (0.1–5.7)	2.7 (0.7–10.2)	-	-	-	-	-	-
Kero [48]	Male partners of pregnant women, 19–46	per 100py	-	-	6.0 (2.9–9.2)	1.2 (0.2–3.4)	-	-	-	-	-	-
Kero [30]^a^	Male partners of pregnant women, 20–52	% at 7 years	14.3 % (NTS) (4.8–30.3)	-	-	-	Prevalent	% at 7 years	100 % (TS) (64.4–100)	-	-	-
Kero [46]^a^	Male partners of pregnant women, 20–52	% at 7 years	69.2 % (TS) (59.5–77.2)	-	-	-	Prevalent	per 100py	3.8 (TS) (1.6–6.1)	-	4.9 (2.3–7.4)	1.4 (0–2.7)
Rintala [50]	Male partners of pregnant women, 20–52	% at 24 m	-	~10 % (NTS, first)	-	-	Prevalent	% at 24 m	-	~5 % (NTS)	-	-
Videla [23]	HIV + ve MSM, 20- > 69 HIV + ve MSW, 20- > 69	per 100py	6.1 (NTS, first) (4.2–8.4) 5.7 (3.0–9.7)	-	-	-	Prevalent	per 100py	19.3 (NTS) (12.8–27.7) 15.8 (7.6–29.0)	-	-	
Darwich [47]	HIV + ve MSM, 20- > 69 HIV + ve MSW, 20- > 69	per 100py	-	-	1.0 (0.4–2.0) 2.6 (1.1–5.4)	0.2 (0–1.0) 0.7 (0.1–2.4)	Prevalent	% at 24 m	48.3 % (TS) (35.2–61.6) 34 % (17.9–54.3)	-	22.7 per 100py (11.3–40.6)	9.1 per 100py (1.8–26.5)
Kreimer [44]	HIV-ve men, 18–70	per 100py	6.7 (NTS, first) (5.5–8.0)	3.0 (2.2–3.8)	1.0 (0.6–1.6)	0.0 (0–0.4)	-	-	-	-	-	-
Women												
Pickard [49]	Female students, 18–30	per 100py	6.8 (TS) (3.7–9.8)	-	-	-	Prevalent	% at 3 m	60.6 (TS) (42.1–77.1)	76.9 (46.2–95.0)-	100 (15.8–100)-	-
D’Souza [15]	HIV-ve women HIV + ve women	per 100py	20.4 (TS) (8.4–44.4) 39.6 (25.2–57.6)	-	-	-	Prevalent	% at 6 m	20.0 (NTS) (0.5–71.6) 40.0 (22.7–59.4)	-	-	-
Kero [30]	Pregnant women, 19–46	% at 7 years	14.3 % (NTS) (5.4–28.5)	-	-	-	Prevalent	% at 7 years	100 % (TS) (29.2–100)	-	-	-
Rintala [50]	Pregnant women, 19–46	% at 24 m	-	~10 % (NTS, first)	-	-	Prevalent	% at 24 m	-	0 % (NTS)	-	-
Men and women												
Kurose [52]	Male/female volunteers, 3–85	-	-	-	-	-	Prevalent	% at 30 m	50.0 (TS) (6.8–93.2)	-	100 (1 sample only)	-

*Abbreviations*: *MSM* men who have sex with men, *MSW* men who have sex with women, *py* person-years, *y* years, *m* months, *TS* type-specific incidence defined as at least one positive test for any HPV type or HR-HPV type not detected at baseline, *NTS* non-type-specific incidence defined as at least one positive test for any HPV type or HR-HPV type among those negative for any HPV DNA or any HR-HPV DNA at baseline

^a^Study [30] considered a subset of men (46/131) included in [46]. Study [30] reported HPV status at baseline and at 7 years, whereas study [46] considered HPV status at baseline and all subsequent follow-up visits (i.e., 2 m, 6 m, 24 m, 36 m, and 7y)

#### Incidence in men

The incidence of oral HPV infection was 5.7–6.1/100 person-years in HIV + ve men and 6.7/100 person-years in HIV-ve men [23, 44]. In another study conducted in university students, oral HPV incidence over 1 year was 12.3 % [45]. Finally, in the Finnish cohort, oral HPV infection was reported in 14.3–69.2 % of male partners of pregnant women 7 years after baseline. The variability in results was due to the varying sampling timetable [30, 46]. In studies reporting type-specific incidence, HPV-16 was the most frequently identified type [44, 45, 47, 48].

Two studies reported age-specific incidence rates of oral infections in men. In one study in university students the age-specific incidence was higher in the 21–24 years age-group than in the 18–20 years age-group [45]. In HIV-ve men there was no increased risk of incident HR-HPV oral infection across age groups (range 18–73 years) [44].

#### Incidence in women

The incidence of oral HPV infections in HIV-ve women was 6.8–20.4/100 person-years versus 39.6/100 person-years in HIV + ve women [15, 49]. In the Finnish cohort, oral infections were present in approximately 10 % of men and women after 24 months follow-up, and in 14.3 % of men and women after 7 years [30, 50]. Incidence rates in women appeared to be similar or higher than rates in men, depending on the study.

#### Clearance and persistence in men and women

Clearance rates of oral infections in men were reported in two study cohorts (Table 6). In HIV + ve men, the clearance rate of prevalent oral infections in MSM was 19.3/100 person-years, or 48.3 % after two-year follow-up [23, 47]. Rates for HIV + ve MSW were 15.8/100 person-years and 34 %, respectively [23, 47]. The time-to-clearance of oral HR-HPV in HIV-ve men was 6.9 months. Time-to-clearance of oral HPV-16 infection was between 7.3 and 37.1 months in HIV-ve men, and was higher (42.3–48.3 months) in HIV + ve men (Fig. 1). In the Finnish cohort, approximately 5 % of oral HR-HPV infections in 19 HR-HPV-positive men had cleared by 24 months [50].

In US studies, clearance of any oral HPV in women was 60.6 % at 3 months [49], and clearance at 6 months was 20.0 % in HIV-ve women and 40.0 % in HIV + ve women [15]. No oral HPV infections had cleared in women in the Finnish cohort at 24 months, whereas clearance at 7 years was 100 % [30, 50]. Among women with persistent cervical HPV infection the time-to-clearance of oral HPV infection was 50.0 months, versus 28.2 months in women without cervical HPV infections (Fig. 1) [51].

Persistence of oral HPV infections was reported for three cohorts (Table 3). In 662 healthy volunteers in Japan, four had a prevalent infection and two had persistent infection with the same HPV-type after 2.5 years [52]. The persistence of prevalent HR-HPV infections in male and female university students was 42 % after three months [49]..

#### Risk factors for non-cervical and oral HR-HPV infection

Penile and/or male genital infections with HR-HPV were significantly associated with younger age in only one study [19], although three articles reported no significant association with age [17, 31, 53]. Other significant risk factors were anal intercourse, HPV status of the partner, number of partners (current or lifetime), and HIV infection [17, 19, 22, 26, 31, 53]. Being uncircumcised was identified as a risk factor for penile HR-HPV infection in one study in HIV-ve men [19], but not in another [28].

An increased likelihood of clearance of HR-HPV penile infection was associated with an increased number of sex partners and circumcision (in HIV-ve men) [19, 31]. One study reported increased clearance in men with self-reported urethral discharge [19], but other sexually-transmitted genital infections were not associated with clearance [19, 31].

Risk factors for anal HR-HPV infection in women were younger age, condom use (possibly due to using the same condom for vaginal and anal sex), higher lifetime number of partners (≥6), and advanced HIV infection and smoking (in HIV + ve women) [36, 37]. There was decreased likelihood of clearance of anal HR-HPV infection in women with a 2–10-pack-year smoking history [39].

No risk factors were significantly associated (multivariate analyses) with anal HR-HPV infection in men.

Risk factors for persistence of oral HR-HPV were only available from one study: a history of smoking was significantly associated with longer persistence whereas a history of genital warts was associated with shorter persistence [46].

## Discussion

The available data suggest a high incidence of non-cervical HPV infections in men and women, with HPV-16 most consistently ranking among the HR-HPV types with the highest incidence rates and the lowest clearance rates in all sites. Despite relatively few studies providing data for cervical and other genital infections in comparable populations, there appears to be a general pattern of both higher incidence and clearance of non-cervical versus cervical genital infections. Around one-half of the identified articles assessed HPV at male penile/other genital sites. There was a wide variation in HPV incidence reported at these sites, although the time-to-clearance was comparable across all studies, with the exception of substantially longer time-to-clearance in HIV + ve men. HIV status, circumcision, number of sex partners and HPV status of the partner significantly influenced HR-HPV incidence, clearance and persistence rates at male anogenital sites in some, but not all studies. Some of these risk factors are the same as those identified for cervical HPV infection, for which the number of sex partners is the most important one [54].

Unlike cervical intraepithelial neoplasia, anal-intraepithelial neoplasia infrequently undergoes malignant transformation in immunocompetent individuals, but the risk of malignant transformation is much greater in HIV + ve individuals [55]. Corresponding with the higher incidence of anal cancer in MSM and HIV + ve men compared to the general population, we also found consistently higher incidences of anal HPV infection in these groups compared to MSW and HIV-ve men, respectively. There was a lack of similar comparison data for these risk groups for clearance. The primary exception was Nyitray et al. [35], which indicated potentially lower clearance rates for MSM versus MSW, but multivariate analyses directly comparing the two groups within the same model were not conducted.

The Hawaiian studies by Goodman et al. showed an incidence of anal HPV infection that was three-times higher than cervical HPV infection [36, 39, 41], although a recent review concluded that the prevalence of anal and cervical infections are similar in women, and that cervical HPV infection is a risk factor for anal HPV infection [56]. As prevalence is determined by the incidence rate multiplied by the average duration of infection, the similar prevalence between anal and cervical infections could be due the higher incidence of anal infections being offset by simultaneously higher clearance. The Hawaiian cohorts provided indication of higher clearance of anal infections, but despite this study having one of the largest, most carefully followed cohorts, there were limited samples sizes to assess clearance rates, especially for individual types. This was in part due to the fact that the analyses of clearance focused on infections that were acquired during the course of the study.

The incidence of oral HPV infection was within the same range in men and women, although the prevalence of oral HPV infection has been reported to be higher in men [57]. Our data were too limited to conclude whether there were differences in persistence or clearance rates between men and women. It is thought that the oncogenicity of HPV is similar in oral and cervical cancers, although numerous gaps remain in understanding the risk factors that influence progression from oral infection to oral neoplasia [57].

The factors that influence oncogenesis at non-cervical sites are not well understood, and appear to differ by site and between populations (including men versus women, immunosuppressed versus immunocompetent, circumcised versus uncircumcised) [57, 58]. Although the incidence of HPV infection at non-cervical sites appears to be high, the increased capacity for clearance of HPV observed may contribute to the lower occurrence of these types of cancers. Overall, data describing the natural history of non-cervical cancers are lacking.

A potential limitation of our study is the non-inclusion of articles in which vaginal swabs were self-collected. The single study that we identified using physician-collected vaginal swabs may not be representative for this site as it was limited to university students. However, it was one of the highest quality studies, following more than 400 women aged 18–20 years old every 4 months for 10 years and prospectively collecting sexual behavior data. The studies on cervical infections were selected from specific non-systematic searches aimed to identify comparable populations with which to compare to non-cervical infections. Thus, the results may not be applicable beyond the study setting. Most studies were not properly designed for risk factor analysis and should be interpreted cautiously.

The studies we identified were substantially different in terms of their design, including follow-up times and sampling intervals which ranged from several months to 7 years, as well as the number of types and methods used for HPV genotyping. Although it is difficult to apply a standard grading to these studies, the highest quality studies tended to be those that followed cohorts of at least 200 subjects and conducted multiple follow-up visits ≤6 months apart for at least one year. At least one such study provided data for each of the sampling sites: penile [17–19, 22, 24, 53], male genital [25, 26, 28, 31, 53, 59], anal [34, 36, 38, 39], vulvovaginal [43], oral [44].

The available published data are limited by the lack of age-specific estimates, and information on clearance/persistence of infection was frequently based on very small sample sizes, resulting in imprecise estimates. Few studies provided incidence or clearance data on oral or anal HPV infections in women versus men, and the relative burden of these infections in each sex is not clear. Specific estimates of incidence and clearance of HPV-16, which is involved in the majority of non-cervical cancers, and HPV-18 which may also be important in some sites, as well as estimation of clearance rates at 6 and 12 months, could inform on potentially important endpoints for clinical trials of vaccine efficacy. Finally, because just a few countries contributed data to this review and given the important differences in study designs, outcomes and populations, it is important to note the limited generalizability of these results to other populations.

## Conclusions

Cases of cervical cancer are projected to increase, emphasising the importance of ongoing efforts to provide access of effective HPV vaccines to all [1, 60]. As yet, the implications of HPV vaccination for prevention of non-cervical cancers have not been fully explored [58]. Some countries have recommended HPV vaccination for young males on the basis that vaccination will prevent HPV-associated cancers in men, as well as theoretical benefits in preventing transmission of HPV to women [61, 62]. Programs targeting prevention of cervical HPV infection could have additional benefits on the non-cervical disease burden. This review suggests that there are parallels between the epidemiology of cervical and non-cervical HPV infections in terms of the incidence and distribution of HPV types, and of risk factors for HPV infection. However, these parallels may not be directly applicable to disease at non-cervical sites. More detailed and extensive studies could provide useful information for estimating vaccine impact, the wider cost-benefit of HPV vaccination, and for guiding vaccination policy.

## Additional files

Additional file 1: Search strings; Article screening; Data extraction; Identification of papers on cervical HPV infection in same/similar study populations; Quality control. (DOCX 139 kb) Additional file 2: Table S1. Summary of HPV testing conducted in 38 articles included in the review. (XLSX 38 kb)
