# Supplementary material for: The incidence, clearance and persistence of non-cervical human papillomavirus infections: a systematic review of the literature
Source: BMC Infect Dis. 2016 Jun 14;16:293. doi: 10.1186/s12879-016-1633-9 (PMC4908763; doi:10.1186/s12879-016-1633-9)
Supplement: Additional file 1: — Search strings; Article screening; Data extraction; Identification of papers on cervical HPV infection in same/similar study populations; Quality control. (DOCX 139 kb) [file 12879_2016_1633_MOESM1_ESM.docx]

**The incidence, clearance and persistence of non-cervical human papillomavirus infections: a systematic review of the literature**

**Supplement**

**Search strings**

The following search strings were used to identify relevant articles registered in PubMed as of July 21st 2014*. The searches for oral infections were done separately for the purposes of a separate review. Three search strings included terms for human papilloma virus, non-cervical (or oral) sites and the outcomes of the review objectives. Within one search string, possible relevant search terms were combined using ‘OR’. Search strings 1, 2 and 3 were run in combination using ‘AND’ (i.e., #1 AND #2 AND #3).

**Non-cervical anogenital infections**

#1: Search string Human Papilloma Virus

(“Papillomaviridae”[Mesh] OR “Papillomavirus infections”[Mesh] OR Human papillomavirus*[tiab] OR “HPV”[tiab] OR “human papilloma”[tiab])

#2: Search string non-cervical sites

(“Anal canal”[Mesh] OR anus[tiab] OR anal[tiab] OR Vulva[Mesh] OR vulva[tiab] OR Vagina[Mesh] OR vagina*[tiab] OR Penis[Mesh] OR penis[tiab] OR penile[tiab])

#3: Search string review objectives 1, 2 and 3

("Incidence"[Mesh] OR incidenc*[tiab] OR persist*[tiab] OR clearance[tiab] OR duration[tiab] OR “Risk factors”[Mesh] OR risk factor*[tiab] OR high-risk group*[tiab] OR “vertical transmission”[tiab] OR “perinatal transmission”[tiab] OR sex[tiab] OR kiss*[tiab] OR “natural history”[tiab] OR “Epidemiology”[Mesh] OR “epidemiology”[subheading] OR epidemiology[tiab] OR precancerous[tiab] OR precursor[tiab] OR intraepithelial[tiab] OR dysplasia*[tiab] OR VIN[tiab] OR VAIN[tiab] OR PIN[tiab] OR PEIN[tiab] OR AIN[tiab])

**Oral infections**

#1: Search string Human Papilloma Virus

(“Papillomaviridae”[Mesh] OR “Papillomavirus infections”[Mesh] OR Human papillomavirus*[tiab] OR “HPV”[tiab] OR “human papilloma”[tiab])

#2: Search string head and neck sites

(“Head and neck neoplasms”[Mesh] OR head and neck neoplasm*[tiab] OR “cancer of head and neck”[tiab] OR “cancer of the head and neck”[tiab] OR head and neck cancer*[tiab] OR head and neck carcinoma*[tiab] OR Oropharynx[Mesh] OR oropharynx[tiab] OR oropharyngeal[tiab] OR Pharynx[Mesh] OR pharyngeal[tiab] OR pharynx[tiab] OR Nasopharynx[Mesh] OR nasopharyngeal[tiab] OR nasopharynx[tiab] OR Hypopharynx[Mesh] OR hypopharyngeal[tiab] OR hypopharynx [tiab] OR Larynx[Mesh] OR laryngeal[tiab] OR larynx[tiab] OR “Paranasal sinuses”[Mesh] OR paranasal sinus*[tiab] OR Esophagus[Mesh] OR esophageal[tiab] OR esophagus[tiab] OR oesophageal[tiab] OR oesophagus[tiab] OR “Salivary glands”[Mesh] OR salivary gland*[tiab] OR Mouth[Mesh] OR mouth[tiab] OR oral[tiab] OR throat[tiab] OR Tongue[Mesh] OR tongue[tiab] OR “Nasal cavity”[Mesh] OR nasal cavity[tiab] OR oral cavity[tiab] OR “Palatine tonsil”[Mesh] OR tonsil*[tiab] OR Lip[Mesh] OR lip[tiab] OR palat*[tiab] OR Gingiva[Mesh] OR gingiva*[tiab])

#3: Search string review objectives 1, 2, 3 and 4

(“Biological markers”[Mesh] OR biological marker*[tiab] OR biomarker*[tiab] OR surrogate marker*[tiab] OR surrogate endpoint*[tiab] OR **precancerous[tiab]** OR **precursor[tiab**] OR **“natural history”[tiab]** OR “**Epidemiology”[Mesh] OR “epidemiology”[subheading] OR epidemiology[tiab]** OR **"Incidence"[Mesh]** OR **incidenc*[tiab]** OR "Prevalence"[Mesh] OR prevalenc*[tiab] OR **persist*[tiab]** OR **clearance[tiab]** OR **duration[tiab]** OR **“Risk factors”[Mesh]** OR **risk factor*[tiab]** OR **high-risk group*[tiab]** OR **“vertical transmission”[tiab]** OR **“perinatal transmission”[tiab]** OR “oral sex”[tiab] OR **kiss*[tiab]** OR **intraepithelial[tiab]** OR **dysplasia[tiab]** OR **LIN[tiab]** OR **NPIN[tiab]** OR **EIN[tiab]** OR **OIN[tiab]** OR neoplasms[Mesh] OR neoplasms[tiab] OR cancer[tiab] OR carcinoma[Mesh] OR carcinoma[tiab] OR tumor[tiab])

*Note: Non-bolded search terms were only used to identify articles registered in PubMed as of April 19th 2013 for purposes of a previous review with additional objectives that are not included in the current manuscript.

**Article screening**

Articles were assessed by title and abstract. Articles that did not contain information relevant to the research objectives were not selected for full-text assessment: these included diagnostic test research, letters to the editor, editorials or comments. In case of doubt, the article was checked full-text in the second selection step.

Articles selected through initial screening were assessed by full-text review according to pre-defined inclusion and exclusion criteria. Articles that did not contain relevant information or contained information of poor quality were excluded. Articles on vaccination were screened for relevant data in the control arm.

**Data extraction**

A standardized checklist (Coordination of Cancer Clinical Practice Guidelines) was used to assess the quality of each study. The checklist was used by the Pallas epidemiologist to avoid selection references with poor reporting or critical quality issues. Predefined aspects of a study were qualitatively scored using - - or -, +/-, + or ++. The checklist was not designed to calculate a total quality score of summed + and - to assess differences in quality between studies. The final decision whether the quality of a study was sufficient or not for inclusion was based on the expertise of the epidemiologist, keeping the results of the checklist and the objectives of the review in mind. In case of doubt, the epidemiologist always discussed the paper with a second epidemiologist at Pallas.

The predefined aspects of the studies that were qualitatively scored included:

- The study addresses an appropriate and clearly focused question
- The outcomes are clearly defined
- The main potential confounders are identified and taken into account in the design and analysis
- The study was done to minimize confounding/bias
- The results of the study are applicable to the patients group targeted in the search question

Determination of risk factors for incident, cleared, and persistent infections

Because the included studies did not measure risk factors in the same way, risk factors that in essence measured the same issue were combined. Only factors of interest that showed significant risk or protection were included. The strength of evidence was rated as follows:

- strong (++): more than one article reported the factor to be significant in a multivariate analysis and there were no articles that found the factor to be not significant.
- good (+): more than one article reported the factor to be significant in the multivariate analysis and at least one article reported the factor to be not significant.
- medium (+/-): only one article reported the factor to be significant in a multivariate analysis.
- weak (-): more than one article reported the factor to be significant in an univariate analyses (no multivariate analysis was performed in these articles).
- very weak (--): only one article reported the factor to be significant with an unadjusted outcome.

**Identification of papers on cervical HPV infection in same/similar study populations**

Articles on cervical HPV infections that used the same or similar cohorts as the cohorts in the articles on non-cervical infections were retrieved using the following steps (step 1 and 2 and 3 where necessary):

1. Search on last name of the first author (‘Last name first author[Author]’) combined with the term ‘human papillomavirus’

2. Search on last name of the last author (‘Last name last author[Author]’) combined with the term ‘human papillomavirus’

3. Search on the name of the institute combined with the term ‘human papillomavirus’

If no article on cervical infections written by the same authors or institution of the article on non-cervical infections was found, we attempted to identify articles on cervical infections for the same country in a comparable population. Since this step (step 4) led in some cases to too many hits, we used steps 5-7 to narrow the number of hits down.

4. Search string tailored to the country combined with the term ‘human papillomavirus’ and the term ‘(cervical OR cervix)’.

5. Limited the search to the words selected in step 4 to title and abstract.

6. If after step 5 the number of hits still exceeded a feasible amount of articles, the term ‘inciden*’ was added.

7. If after step 6 the number of hits still exceeds a feasible amount of articles or only provided non-relevant hits, a specific term for the population of interest was added (e.g. young women).

**Quality control**

The first 30% of titles and abstracts were screened in duplicate by two independent researchers from Pallas. The results were compared and discussed before the remaining references were assessed by one researcher.

The first 10% of full text articles were critically appraised in duplicate by two independent researchers from Pallas. The results were compared and discussed early in the process. Any disagreements were adjudicated by a third researcher if necessary. Data extraction: the evidence tables were compiled by junior researchers and reviewed by the senior researcher of the project.

Reported estimates and conversions were re-checked by ST.

**Additional file 1 - Table 1:** Summary of HPV testing conducted in 38 articles included in the review

| **Author** | **Assay** | **HPV types** |
| --- | --- | --- |
| Tobian [1] | Roche Linear Array | **14 HR**: 16, 18, 31, 33, 35, 39, 45, 51, 52, 56, 58, 59, 66, 68 |
| Kjaer [2] | GP05+/06+ | **16 HR**: 16, 18, 31, 33, 35, 39, 45, 51, 52, 56, 58, 59, 66, 68, 73, 82  LR: 6, 11, 26, 34, 40, 42, 43, 44, 53, 54, 55, 57, 61, 70, 71, 72, 81, 83, 84, 89. |
| Backes [3] | GP05+/06+ | **14 HR**: 16, 18, 31, 33, 35, 39, 45, 51, 52, 56, 58, 59, 66, 68  LR: 6, 11, 26, 32, 34, 40, 42, 43, 44, 53, 54, 55, 57, 61, 64, 67, 69, 70, 71, 72, 73, 81, 83, 84, 85, 86, 89, JC9710, X. |
| Mbulawa [4] | Roche Linear Array | **18 HR**: 16, 18, 26, 31, 33, 35, 39, 45, 51, 52, 53, 56, 58, 59, 66, 68, 73, 82  LR: 6, 11, 26, 40, 42, 54, 55, 61, 62, 64, 66, 67, 69, 70, 71, , 81, 83, 84, CP6108, IS39 |
| Videla [5] | QIamp/IVD-CE F | **13 HR**: 16, 18, 31, 33, 35, 39, 45, 51, 52, 56, 58, 59, 68  LR :6, 11 |
| Wikstrom [6] | PCR using GP+ | **7 HR**: 16, 18, 31, 33, 45, 56, 73  LR: 6, 11, 40, 42, 43, 44, 54, 62, 66, 67, 70, L11353, CP8304, CP6108 |
| Gray [7] | Roche Linear Array | **14 HR**: 16, 18, 31, 33, 35, 39, 45, 51, 52, 56, 58, 59, 66, 68  LR: 6, 11, 26, 40, 42, 43, 53- 55, 61, 67, 70- 73, 81-84, 108 |
| Serwadda [8] | Roche Linear Array | **14 HR**: 16, 18, 31, 33, 35, 39, 45, 51, 52, 56, 58, 59, 66, 68  LR: 6, 11, 26, 40, 42, 43, 53-55, 61, 6770-73, 81-84, CP6108 |
| Grabowski [9] | Roche Linear Array | **14 HR**: 16, 18, 31, 33, 35, 39, 45, 51, 52, 56, 58, 59, 66, 68  LR: 6, 11, 26, 40, 42, 54, 55, 61, 62, 64, 67, 69, 70, 71-73, 81-84, CP6108, IS39 |
| Giuliano [10] | Roche Linear Array | **13 HR**: 16, 18, 31, 33, 35, 39, 45, 51, 52, 56, 58, 59, 66.  LR: 6, 11, 26, 40, 42, 53-55, 61, 62, 64, 67-73, 81-84, CP6108, IS39 |
| Darwich [11] | QIAamp/IVD-CE F | **13 HR**: 16, 18, 31, 33, 35, 39, 45, 51, 52, 56, 58, 59, 68  LR :6, 11 |
| de Pokomandy [12] | PGMY09/11 | **15 HR**: 16, 18, 31, 33, 35, 39, 45, 51, 52, 56, 58, 59, 66, 68, 82  LR: 6, 26, 34, 40, 42, 44, 51, 53, 54, 61, 62, 67, 69-73, 81, 83, 84, 89 |
| Nyitray [13] | PCR using DNA biotin labelled probes | **13 HR**: 16, 18, 31, 33, 35, 39, 45, 51, 52, 56, 58, 59, 68  LR: 6, 11, 26, 40, 42, 51, 53–55, 61, 62, 64, 66, 67, 69–73, 81-84, 89 |
| Goodman [14], Shvetsov [15] | modified PGMY09/11, | **18 HR**: 16, 18, 31, 33, 35, 39, 45, 51, 52, 53, 56, 58, 59, 66, 68, 70, 73, 82  LR: 6, 11, 42, 54, 61, 72, 84, CP6108, 55, 62, 67, 71, 83, 84 |
| Moscicki [16] | PGMY09/11 | **13 HR**: 16, 18, 31, 33, 35, 45, 51, 52, 56, 68, 69, 73, 82  LR: 2, 6, 11, 42, 44, 54, 55, 57, 61, 62, 64, 67, 69, 70, 71, 72, 81, 83, 84 |
| Mullins [17] | PGMY09/11/HMB01 | **11 HR**: 16, 18, 31/33/35, 39, 45, 51, 52, 56, 58, 59/68/70  LR: 6, 11, 42, 44 |
| Glick [18] | QIAamp liquid bead microarray | **13 HR**: 16, 18, 31, 33, 35, 39, 45, 51, 52, 56, 58, 59, 68  LR: 6, 11, 26, 40, 42, 53, 54, 55, 61, 62, 64, 66, 67, 69-73, 81-84, IS39, cp6108 |
| Hernandez [19] | MY09/MY11 | **15 HR**: 16, 18, 31, 33, 35, 39, 45, 51, 52, 56, 58, 59, 68, 73, 82.  LR: 6, 11, 26, 32, 40, 53, 54, 55, 61, 66, 68, 69, 70, 73, AE2, Pap 155, and Pap 291 (+ mix of 2, 13, 34, 42, 57, 62, 64, 67, 72, W13B). |
| Lu [20] | PGMY09/11 L1 | **13 HR**: 16, 18, 31, 33, 35, 39, 45, 51, 52, 56, 58, 59, 66  LR: 6, 11, 26, 40, 42, 53-55, 61, 62, 64, 67-73, 81-84, CP6108, IS39 |
| Hernandez [21] | PGMY09/11 | **13 HR**: 16, 18, 31, 33, 35, 39, 45, 51, 52, 56, 58, 59, and 68  LR: 6, 11, 26, 40, 42, 53, 54, 55, 61, 62, 64, 66, 67, 69, 70, 71, 72, 73, 81, 82,  83, 84, IS39, CP6109 (HPV-89) |
| Giuliano [22] | PGMY09/11 L1 | **13 HR**: 16, 18, 31, 33, 35, 39, 45, 51, 52, 56, 58, 59, 66  LR: 6, 11, 26, 40, 42, 53-55, 61, 62, 64, 67-73, 81-84, CP6108, IS39 |
| Morales [23] | PG/MY L1 | **13 HR**: 16, 18, 31, 33, 35, 39, 45, 51, 52, 56, 58, 59, 66  LR: 6, 11, 26, 40, 42, 53-55, 61, 62, 64, 66, 67, 69-73, 81-84, IS39, CP6108 |
| Partridge [24] | PGMY09/11 | **19 HR**: 16, 18, 26, 31, 33, 35, 39, 45, 51–53, 56, 58, 59, 66–68, 73, 82  LR: 6, 11, 40, 42, 54, 55, 57, 61, 62, 64, 69–72, 81, 83, 84, CP6108. |
| Lajous [25] | BGH 20/ BPCO4 | **16 HR**: 16, 18, 31, 33, 35, 39, 45, 51, 52, 53, 56, 58, 59, 68, 73, 82  LR: 6, 11, 26, 40, 42, 54, 55, 57, 66, 83, 84 |
| Albero [26] | PGMY09/11 | **13 HR**: 16, 18, 31, 33, 35, 39, 45, 51, 52, 56, 58, 59 68.  LR: 6, 11, 26, 40, 42, 53, 54, 55, 61, 62, 64, 66, 67, 69, 70, 71, 72, 73, 81, 82, 83, 84, IS39, 89 |
| Winer [27] | MY09/11/HMB01 | **5 HR**: 16, 18, 31, 45, 56  LR: 6, 11 |
| Edelstein [28] | MY09/11/HMB01 | **18 HR:** 16, 18, 31, 33, 35, 39, 45, 51, 52, 53, 56, 58, 59, 66, 68, 70, 73, 82  LR: 6, 11, 26, 40, 42, 54, 55, 61, 62, 64, 67, 69, 71, 72, 81, 83, 84, IS39, CP6108 |
| Kero [29] | MY09/11 and GP05+/06+ | **14 HR**: 16, 18, 31, 33, 35, 39, 45, 51, 52, 58, 59, 68, 73, 82.  LR: 6, 11, 42-44, 70 |
| Kero [30] | MY09/11 and GP05+/06+ | **18 HR**: 16, 18, 26, 31, 33, 35, 39, 45, 51, 52, 53, 56, 58, 59, 66, 68, 73, 82  LR: 6, 11, 42-44, 70 |
| Kero [31] | MY09/11 and GP05+/06+ | **18 HR**: 16, 18, 26, 31, 33, 35, 39, 45, 51, 52, 53, 56, 58, 59, 66, 68, 73, 82  LR: 6, 11, 42-44, 70 |
| Louvanto [32] | GP05+/06+ | **18 HR**: 16, 18, 26, 31, 33, 35, 39, 45, 51, 52, 53, 56, 58, 59, 66, 68, 73, 82  LR: 6, 11, 42-44, 70 |
| Rintala [33] | MY09/11 and GP05+/06+ | **12 HR**: 16, 18, 31, 33, 35, 39, 45, 51, 52, 54, 56, 58  LR: 6, 11, 42, 43, 44, 70; HR-HPV: 16, 18, 26, 31, 33, 35, 39, 45, 51, 52, 53, 56, 58, 59, 66, 68, 73, 82 |
| Pickard [34] | PGMY09/11 | **18 HR**: 16, 18, 26, 31, 33, 35, 39, 45, 51, 52, 53, 56, 58, 59, 66, 68, 73, 82  LR: 6, 11, 26, 40, 42, 54, 55, 61, 62, 64, 66, 67, 69, 70-73, 81, 83, 84, IS39, CP6108 |
| D’Souza [35] | PGMY09/11 Roche linear array | **22 HR:** 16, 18, 26, 31, 33, 35, 39, 45, 51, 52, 53, 56, 58, 59, 66, 67, 68, 69, 70, 73, 82, 83  LR (detected): 6, 11, 40, 42, 54, 55, 57, 61, 62, 64, 71, 72, 81, 84, 89 |
| Kurose [36] | MY09/11 L1 | Not listed |
| Darwich [37] | QIAamp/IVD-CE F | **13 HR**: 16, 18, 31, 33, 35, 39, 45, 51, 52, 56, 58, 59, 68  LR :6, 11 |
| Kreimer [38] | Roche Linear Array | **13 HR**: 16, 18, 31, 33, 35, 39, 45, 51, 52, 56, 58, 59, 68.  LR: 6, 11, 26, 40, 42, 53, 54, 55, 61, 62, 64, 66, 67, 69, 70, 71, 72, 73, 81, 82, 83, 84, CP6108, IS39 |

HR = high-risk HPV, LR = low risk HPV. All testing was for HPV DNA

**References**

1. Tobian AAR, Kigozi G, Gravitt PE, Xiao C, Serwadda D, Eaton KP, et al. Human papillomavirus incidence and clearance among HIV-positive and HIV-negative men in sub-Saharan Africa. AIDS. 2012;26:1555–65.

2. Kjaer SK, Munk C, Winther JF, Jørgensen HO, Meijer CJLM, van den Brule AJC. Acquisition and persistence of human papillomavirus infection in younger men: a prospective follow-up study among Danish soldiers. Cancer Epidemiol. Biomarkers Prev. 2005;14:1528–33.

3. Backes DM, Snijders PJF, Hudgens MG, Bailey RC, Bogaarts M, Agot K, et al. Sexual behaviour and less frequent bathing are associated with higher human papillomavirus incidence in a cohort study of uncircumcised Kenyan men. Sex Transm Infect. 2013;89:148–55.

4. Mbulawa ZZA, Marais DJ, Johnson LF, Coetzee D, Williamson A-L. Impact of human immunodeficiency virus on the natural history of human papillomavirus genital infection in South African men and women. J. Infect. Dis. 2012;206:15–27.

5. Videla S, Darwich L, Cañadas M-P, Coll J, Piñol M, García-Cuyás F, et al. Natural history of human papillomavirus infections involving anal, penile, and oral sites among HIV-positive men. Sex Transm Dis. 2013;40:3–10.

6. Wikström A, Popescu C, Forslund O. Asymptomatic penile HPV infection: a prospective study. Int J STD AIDS. 2000;11:80–4.

7. Gray RH, Serwadda D, Kong X, Makumbi F, Kigozi G, Gravitt PE, et al. Male circumcision decreases acquisition and increases clearance of high-risk human papillomavirus in HIV-negative men: a randomized trial in Rakai, Uganda. J. Infect. Dis. 2010;201:1455–62.

8. Serwadda D, Wawer MJ, Makumbi F, Kong X, Kigozi G, Gravitt P, et al. Circumcision of HIV-infected men: effects on high-risk human papillomavirus infections in a randomized trial in Rakai, Uganda. J. Infect. Dis. 2010;201:1463–9.

9. Grabowski MK, Gray RH, Serwadda D, Kigozi G, Gravitt PE, Nalugoda F, et al. High-risk human papillomavirus viral load and persistence among heterosexual HIV-negative and HIV-positive men. Sex Transm Infect. 2014;90:337–43.

10. Giuliano AR, Lee J-H, Fulp W, Villa LL, Lazcano E, Papenfuss MR, et al. Incidence and clearance of genital human papillomavirus infection in men (HIM): a cohort study. Lancet. 2011;377:932–40.

11. Darwich L, Cañadas M-P, Videla S, Coll J, Molina-López RA, Sirera G, et al. Prevalence, clearance, and incidence of human papillomavirus type-specific infection at the anal and penile site of HIV-infected men. Sex Transm Dis. 2013;40:611–8.

12. de Pokomandy A, Rouleau D, Ghattas G, Vézina S, Coté P, Macleod J, et al. Prevalence, clearance, and incidence of anal human papillomavirus infection in HIV-infected men: the HIPVIRG cohort study. J. Infect. Dis. 2009;199:965–73.

13. Nyitray AG, Carvalho da Silva RJ, Baggio ML, Smith D ’elle, Abrahamsen M, Papenfuss M, et al. Six-month incidence, persistence, and factors associated with persistence of anal human papillomavirus in men: the HPV in men study. J. Infect. Dis. 2011;204:1711–22.

14. Goodman MT, Shvetsov YB, McDuffie K, Wilkens LR, Zhu X, Ning L, et al. Acquisition of anal human papillomavirus (HPV) infection in women: the Hawaii HPV Cohort study. J. Infect. Dis. 2008;197:957–66.

15. Shvetsov YB, Hernandez BY, McDuffie K, Wilkens LR, Zhu X, Ning L, et al. Duration and clearance of anal human papillomavirus (HPV) infection among women: the Hawaii HPV cohort study. Clin. Infect. Dis. 2009;48:536–46.

16. Moscicki A-B, Ma Y, Farhat S, Jay J, Hanson E, Benningfield S, et al. Natural history of anal human papillomavirus infection in heterosexual women and risks associated with persistence. Clin. Infect. Dis. 2014;58:804–11.

17. Mullins TLK, Wilson CM, Rudy BJ, Sucharew H, Kahn JA. Incident anal human papillomavirus and human papillomavirus-related sequelae in HIV-infected versus HIV-uninfected adolescents in the United States. Sex Transm Dis. 2013;40:715–20.

18. Glick SN, Feng Q, Popov V, Koutsky LA, Golden MR. High rates of incident and prevalent anal human papillomavirus infection among young men who have sex with men. J. Infect. Dis. 2014;209:369–76.

19. Hernandez AL, Efird JT, Holly EA, Berry JM, Jay N, Palefsky JM. Incidence of and risk factors for type-specific anal human papillomavirus infection among HIV-positive MSM. AIDS. 2014;28:1341–9.

20. Lu B, Wu Y, Nielson CM, Flores R, Abrahamsen M, Papenfuss M, et al. Factors associated with acquisition and clearance of human papillomavirus infection in a cohort of US men: a prospective study. J. Infect. Dis. 2009;199:362–71.

21. Hernandez BY, Shvetsov YB, Goodman MT, Wilkens LR, Thompson P, Zhu X, et al. Reduced clearance of penile human papillomavirus infection in uncircumcised men. J. Infect. Dis. 2010;201:1340–3.

22. Giuliano AR, Lu B, Nielson CM, Flores R, Papenfuss MR, Lee J-H, et al. Age-specific prevalence, incidence, and duration of human papillomavirus infections in a cohort of 290 US men. J. Infect. Dis. 2008;198:827–35.

23. Morales R, Parada R, Giuliano AR, Cruz A, Castellsagué X, Salmerón J, et al. HPV in female partners increases risk of incident HPV infection acquisition in heterosexual men in rural central Mexico. Cancer Epidemiol. Biomarkers Prev. 2012;21:1956–65.

24. Partridge JM, Hughes JP, Feng Q, Winer RL, Weaver BA, Xi L-F, et al. Genital human papillomavirus infection in men: incidence and risk factors in a cohort of university students. J. Infect. Dis. 2007;196:1128–36.

25. Lajous M, Mueller N, Cruz-Valdéz A, Aguilar LV, Franceschi S, Hernández-Avila M, et al. Determinants of prevalence, acquisition, and persistence of human papillomavirus in healthy Mexican military men. Cancer Epidemiol. Biomarkers Prev. 2005;14:1710–6.

26. Albero G, Castellsagué X, Lin H-Y, Fulp W, Villa LL, Lazcano-Ponce E, et al. Male circumcision and the incidence and clearance of genital human papillomavirus (HPV) infection in men: the HPV Infection in men (HIM) cohort study. BMC Infect. Dis. 2014;14:75.

27. Winer RL, Lee S-K, Hughes JP, Adam DE, Kiviat NB, Koutsky LA. Genital human papillomavirus infection: incidence and risk factors in a cohort of female university students. Am. J. Epidemiol. 2003;157:218–26.

28. Edelstein ZR, Schwartz SM, Hawes S, Hughes JP, Feng Q, Stern ME, et al. Rates and determinants of oral human papillomavirus infection in young men. Sex Transm Dis. 2012;39:860–7.

29. Kero K, Rautava J, Syrjänen K, Grenman S, Syrjänen S. Oral mucosa as a reservoir of human papillomavirus: point prevalence, genotype distribution, and incident infections among males in a 7-year prospective study. Eur. Urol. 2012;62:1063–70.

30. Kero KM, Rautava J, Syrjänen K, Kortekangas-Savolainen O, Grenman S, Syrjänen S. Stable marital relationship protects men from oral and genital HPV infections. Eur. J. Clin. Microbiol. Infect. Dis. 2014;33:1211–21.

31. Kero K, Rautava J, Syrjänen K, Willberg J, Grenman S, Syrjänen S. Smoking increases oral HPV persistence among men: 7-year follow-up study. Eur. J. Clin. Microbiol. Infect. Dis. 2014;33:123–33.

32. Louvanto K, Rautava J, Syrjänen K, Grénman S, Syrjänen S. The clearance of oral high-risk human papillomavirus infection is impaired by long-term persistence of cervical human papillomavirus infection. Clin. Microbiol. Infect. 2014;20:1167–72.

33. Rintala M, Grénman S, Puranen M, Syrjänen S. Natural history of oral papillomavirus infections in spouses: a prospective Finnish HPV Family Study. J. Clin. Virol. 2006;35:89–94.

34. Pickard RKL, Xiao W, Broutian TR, He X, Gillison ML. The prevalence and incidence of oral human papillomavirus infection among young men and women, aged 18-30 years. Sex Transm Dis. 2012;39:559–66.

35. D’Souza G, Fakhry C, Sugar EA, Seaberg EC, Weber K, Minkoff HL, et al. Six-month natural history of oral versus cervical human papillomavirus infection. Int. J. Cancer. 2007;121:143–50.

36. Kurose K, Terai M, Soedarsono N, Rabello D, Nakajima Y, Burk RD, et al. Low prevalence of HPV infection and its natural history in normal oral mucosa among volunteers on Miyako Island, Japan. Oral Surg Oral Med Oral Pathol Oral Radiol Endod. 2004;98:91–6.

37. Darwich L, Cañadas MP, Videla S, Coll J, Molina-López RA, Cobarsi P, et al. Oral human papillomavirus type-specific infection in HIV-infected men: a prospective cohort study among men who have sex with men and heterosexual men. Clin. Microbiol. Infect. 2014;20:O585–9.

38. Kreimer AR, Pierce Campbell CM, Lin H-Y, Fulp W, Papenfuss MR, Abrahamsen M, et al. Incidence and clearance of oral human papillomavirus infection in men: the HIM cohort study. Lancet. 2013;382:877–87.
